# Factor Structure of the Brief Coping Orientation to Problems Experienced Inventory (Brief-COPE) in Chinese Nursing Students

**DOI:** 10.3390/nursrep15020046

**Published:** 2025-01-29

**Authors:** Cheng Cheng, Qingling Wang, Jie Bai

**Affiliations:** 1 School of Nursing, Fudan University, Shanghai 200437, China; 2 School of Nursing and Health Management, Shanghai University of Medicine and Health Sciences, Shanghai 201318, China; wangql@sumhs.edu.cn; 3 Shanghai First Maternity and Infant Hospital, School of Medicine, Tongji University, Shanghai 200070, China; baijiebbmc@sina.com

**Keywords:** coping, psychometric properties, nursing, student

## Abstract

**Background/Objectives**: Coping strategies are influenced by sociocultural factors, and an understanding of how the Brief-COPE functions within the Chinese student population is important for its validity and reliability. This study aimed to explore the factor structure of the Brief Coping Orientation to Problems Experienced Inventory (Brief-COPE) in Chinese nursing students. **Methods**: A cross-sectional study design was adopted. A total of 284 college nursing students, aged 18 years or older, were recruited from a medical university in China using convenience sampling. Exploratory factor analysis (EFA) was conducted to identify the underlying domain structure of the Brief-COPE within those students. This study adhered to the Strengthening the Reporting of Observational Studies in Epidemiology (STROBE) Statement. **Results**: The Brief-COPE demonstrated robust validity, revealing eight distinct factors: positive reframing coping, avoidant and passive coping, seeking social support, self-blame and emotional distress coping, denial and deflective coping, spirituality and humor coping, avoidance and emotional release coping, and adaptive acceptance with distraction. The scale exhibited good internal consistency, as indicated by a Cronbach’s alpha of 0.759. **Conclusions**: The Brief-COPE is a valid tool for assessing coping strategies in Chinese nursing students. Nursing educators could benefit from training aimed at enhancing the use of appropriate strategies. Also, culturally tailored interventions, such as peer support groups and mentorship programs, could further promote coping skills and improve the emotional well-being of these students.

## 1. Introduction

Nursing students often face significant stress due to the demanding nature of their academic and clinical training [1]. The literature shows that nursing students experience more stress compared with students in other disciplines [2]. The common stressors of nursing students included academic stress such as workload, testing, and evaluations; clinical work such as fear of failure in training; emotional responses to patient suffering; communication with patients and colleagues; and social factors such as family responsibilities and financial difficulties [3]. In their first year as registered nurses, they may face challenges such as navigating the healthcare system and using medical devices, fear of interacting with new patients, and difficulty applying policies and procedures. The transition to real-world practice can be overwhelming [4]. Nursing educators need to know the prevalence, causes, and levels of stress among students, which not only affect their health but also their academic achievements at different points of time of their study period. Research showed that stress was associated negatively with students’ physical and mental health [5], learning performance [6], and life satisfaction [7].

Coping refers to the cognitive and behavioral efforts individuals employ to manage the internal and external demands of stressful situations [8]. It plays a pivotal role in determining how people adapt to challenges, whether those challenges stem from personal, academic, or professional environments. Coping strategies can vary widely and are typically categorized into problem-based and emotion-based. In high-stress fields like healthcare and education, effective coping is particularly critical. It helps individuals maintain mental and emotional balance, improve performance, and build resilience [9,10]. The ability to cope effectively is influenced by factors such as personality, cultural background, available resources, and the specific nature of stressors encountered. For nursing students, who navigate intense academic demands, clinical pressures, and emotional challenges, coping strategies are essential [11]. Understanding how individuals manage stress not only provides insights into their psychological well-being but also informs the development of interventions to support mental health, improve learning outcomes, and promote long-term professional success. Developing effective coping strategies is vital for maintaining their mental health, preventing burnout, and achieving success in both academic and clinical settings [12]. Evidence from meta-analyses indicated that coping-based interventions, such as mindfulness, significantly reduce levels of depression, anxiety, and stress in nursing students [13]. In addition, another meta-analysis on stress and coping interventions for nursing students found that these approaches effectively reduce psychological and physiological stress, including decreases in systolic blood pressure (SBP), heart rate (HR), and cortisol levels [14]. The ability to manage stress not only affects nursing students’ current well-being and academic performance but also plays a critical role in shaping their future as competent and resilient healthcare professionals in health crises such as COVID-19 [15]. Therefore, understanding and supporting nursing students’ coping mechanisms is essential for enhancing their overall learning experience and long-term well-being.

While various coping strategy instruments are available, such as Folkman and Lazarus’s Ways of Coping Questionnaire [16], the Multidimensional Coping Inventory [17], and the Medical Coping Modes Questionnaire [18], the length of these tools often makes them less suitable for use in extensive research protocols or clinical studies. In contrast, the 28-item Brief-COPE offers a more concise alternative and has been widely utilized in numerous studies. The Brief-COPE (Coping Orientation to Problems Experienced) inventory is a widely recognized and extensively used tool for assessing the coping strategies individuals employ in response to stress [19]. Developed as a shortened version of the original COPE inventory [20], the Brief-COPE provides a comprehensive yet concise assessment of both positive and negative coping strategies [19]. With 28 items divided into 14 scales, it measures a range of coping behaviors, including active coping, emotional support, denial, and self-blame. Its ability to capture diverse coping responses makes it an effective tool for understanding how individuals, including nursing students, cope with stress. The Brief-COPE has been used in various populations and settings worldwide, offering insights into the coping mechanisms individuals adopt in different contexts [21]. However, for it to be applied effectively in specific populations, it is crucial to ensure its and reliability and validity. Moreover, the validation of the Brief-COPE has yielded inconsistent results, largely due to the complex and multifaceted nature of coping across different stressors and populations.

While the Brief-COPE has been used a lot in several student populations in Spain [22], Japan [23], the US [24], and Malaysia [25], there is a gap in research regarding its use with Chinese nursing students. The coping strategies employed by nursing students in China may differ from those in Western or other cultural settings due to different settings and cultural values. Therefore, it is essential to validate the Brief-COPE in the Chinese context to ensure that the tool accurately reflects the coping mechanisms that Chinese nursing students employ.

In China, students may experience stress from the educational system. To obtain more resources, most Chinese schools try to improve the average academic performance of students by assigning many assignments, administering mock tests, and requiring long study hours and remedial work [26]. Additionally, graduate employment is affected by the massification of higher education [27]. These stressors require the development of coping strategies tailored to their specific challenges. Moreover, cultural values such as collectivism, filial piety, and an emphasis on modesty and harmony are likely to influence how Chinese nursing students perceive and respond to stress.

Understanding the coping mechanisms of Chinese nursing students is critical. By addressing their mental health and well-being, the findings may contribute to fostering coping and preparing students to thrive in challenging healthcare environments, ultimately ensuring a healthier and more effective nursing workforce. Insights into their coping strategies can inform educational and institutional policies aimed at promoting effective coping mechanisms, reducing stress, and enhancing both academic and clinical performance. Therefore, this study aimed to address this gap by validating the psychometric properties of the Brief-COPE for Chinese nursing students, ensuring its cultural relevance and practical applicability.

## 2. Materials and Methods

### 2.1. Study Design

This study utilized a cross-sectional design. This study was conducted in adherence to the Strengthening the Reporting of Observational Studies in Epidemiology (STROBE) Statement.

### 2.2. Sample

Participants for this study were recruited from a nursing school at a medical university in Eastern China. Eligible participants included nursing students currently enrolled in undergraduate or graduate nursing programs. The inclusion criteria required participants to be at least 18 years old and to have completed a minimum of one year of nursing education, ensuring exposure to both academic and clinical training stressors. Exclusion criteria were nursing students who were not enrolled in full-time education, were not native Chinese speakers, or had a history of significant mental health disorders (e.g., severe anxiety or depression) that could interfere with their coping responses.

### 2.3. Sample Size

Comrey and Lee [28] proposed a general rating scale for assessing sample sizes in factor analysis, categorizing 100 as poor, 200 as fair, 300 as good, 500 as very good, and 1000 or more as excellent. However, establishing a universal minimum sample size is challenging, as the required sample size depends on variables such as the number of items and the number of factors being analyzed. For this study, the widely accepted “rule of thumb” of having 10 participants per item in the instrument was applied [29]. Accordingly, a total sample size of 280 participants was calculated, with an additional 15% added to account for potential attrition.

### 2.4. Measures

Socio-demographic data were collected, including age, gender, health status, and academic year.

The Brief-COPE (Coping Orientation to Problems Experienced) is a widely recognized instrument designed to assess the coping strategies individuals use in response to stress [19]. The original Brief-COPE comprises 28 items distributed across 14 subscales, capturing a variety of coping strategies ranging from active coping and planning to self-blame and denial. Each item is scored on a 4-point Likert scale, ranging from 1 (“I usually do not do this at all”) to 4 (“I usually do this a lot”), with higher scores reflecting more frequent use of the respective coping strategy. The Brief-COPE has been previously applied to Chinese populations with chronic illnesses and has demonstrated satisfactory reliability [30].

### 2.5. Data Collection

The questionnaire was delivered to participants via an online survey platform to enhance convenience and accessibility. Before participation, students were informed about the study through an advertisement posted on the nursing program community site, which included details about the purpose of the study, the voluntary nature of participation, and assurance of confidentiality. Clear instructions were provided to guide participants through the survey, and they were assured that their responses would remain anonymous. Participants were asked to complete the questionnaire in a single sitting, with an estimated completion time of 10–15 min.

### 2.6. Data Analysis

Data analysis was conducted using IBM SPSS version 20.0 (IBM Corp., Armonk, NY, USA). The psychometric properties of the Brief-COPE, including factor structure and internal consistency, were examined. To identify the underlying factor structure of the Brief-COPE inventory for Chinese nursing students, Exploratory Factor Analysis (EFA) was conducted. The EFA was performed using principal component analysis with Varimax rotation. The Kaiser–Meyer–Olkin (KMO) measure and Bartlett’s Test of Sphericity were performed to assess the suitability of the data for exploratory factor analysis. Factors with eigenvalues greater than one were retained, and a scree plot was examined to determine the point of the last significant change in its shape. Items with loadings above 0.4 were included in specific factors, while items with lower loadings were assigned to conceptually appropriate factors. Reliability was assessed through internal consistency, measured by Cronbach’s alpha. This metric evaluates the extent to which items collectively measure the same construct. All statistical tests were two-tailed, with a significance threshold set at 0.05.

## 3. Results

### 3.1. Demographic Characteristics of the Sample

A total of 325 nursing students were approached, of whom 297 agreed to participate. A total of 13 questionnaires were excluded due to incomplete responses, resulting in a final sample of 284 nursing students. The sample included an unbalanced representation of genders, with approximately 9.2% male (*n* = 26) and 90.8% female participants (*n* = 258). Participants’ ages ranged from 18 to 25 years, with the majority (80%) falling between 21 and 24 years. Most participants (88%, *n* = 250) were in the early stages of their nursing education (second year), while 12% (*n* = 34) were at more advanced academic levels (third and fourth years). All students reported that they had no chronic illnesses.

### 3.2. Factor Analysis Results

**Figure 1:**
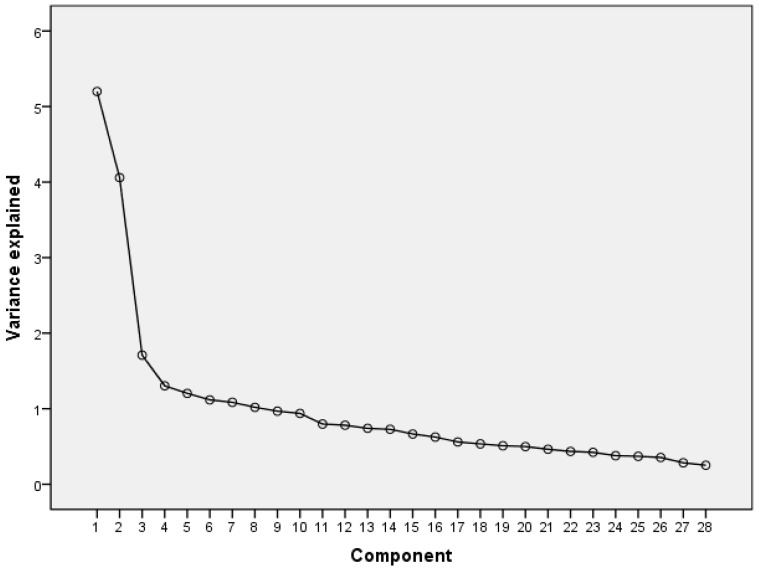
Scree plot.

The KMO value was 0.819, and Bartlett’s Test of Sphericity statistics was 1910.909 (*p* < 0.001), supporting the sampling adequacy and factorability. Our results revealed eight factors with eigenvalues greater than 1.0, accounting for 59.6% of the variance. Table 1 presents the factor loadings of the eight-factor solution, and Figure 1 shows the scree plot.

Each factor was analyzed and named based on the content and thematic alignment of its items.

#### 3.2.1. Positive Reframing Coping

This factor included the subscales of planning (Items 25 and 14), active coping (Items 7 and 2), and positive reframing (Items 17 and 12), and one item from acceptance (Item 24). It was named “positive reframing coping” as it encompasses strategies that actively address the problem through planning and problem-solving while cognitively reframing the situation to identify positive aspects or alternative perspectives.

#### 3.2.2. Avoidant and Passive Coping

This factor comprised the subscale of substance use (Items 1 and 4), one item from religion (Item 2), and one item from behavioral disengagement (Item 16). It was named “avoidant and passive coping” as it reflects strategies that emphasize avoidance or disengagement from the stressor.

#### 3.2.3. Seeking Social Support

This factor included the subscales of instrumental support (Items 23 and 10) and emotional support (Items 15 and 5). It was named “seeking social support” as it represents strategies involving reaching out to others for emotional reassurance or practical assistance.

#### 3.2.4. Self-Blame and Emotional Distress Coping

This factor included the subscale of self-blame (Items 13 and 26), one item from venting (Item 21), and one item from behavioral disengagement (Item 6). It was named “self-blame and emotional distress coping” because it reflects internalized negativity, such as self-blame or criticism, combined with emotional venting and expressions of distress without constructive problem-solving.

#### 3.2.5. Denial and Deflective Coping

This factor included the subscale of denial (Items 3 and 28) and one item from humor (Item 8). It was named “denial and deflective coping” as it represents strategies involving the refusal to acknowledge the reality of a situation (denial) and the use of humor to deflect emotional pain or avoid direct confrontation with the stressor.

#### 3.2.6. Spirituality and Humor Coping

This factor included one item from religion (Item 27) and one item from humor (Item 18). It was named “spirituality and humor coping” as it combines spiritual practices, such as prayer or meditation, with humor to manage stress and build emotional resilience.

#### 3.2.7. Avoidance and Emotional Release Coping

This factor included one item from self-distraction (Item 1) and one item from venting (Item 9). It was named “avoidance and emotional release coping” as it reflects strategies that involve temporary disengagement (e.g., through activities or distractions) and venting of emotions to release tension while processing feelings.

#### 3.2.8. Adaptive Acceptance with Distraction

The final factor included one item from self-distraction and one item from acceptance. It was named “adaptive acceptance with distraction” as it represents a balanced coping strategy that involves accepting the situation as reality while using distractions (e.g., hobbies or entertainment) to reduce emotional intensity. This approach blends emotional adaptation with temporary mental reprieve.

### 3.3. Reliability Results

The Cronbach’s alpha for the Brief-COPE was 0.759, while the values for the eight-factor structure ranged from 0.622 to 0.834, meeting acceptable criteria [31]. Additionally, the corrected item–total correlation coefficients for each item in the Brief-COPE ranged from 0.22 to 0.64, indicating homogeneity among the items. The results for internal consistency, represented by Cronbach’s alpha and item analysis, are shown in Table 2.

## 4. Discussion

To the best of our knowledge, this study represents the first attempt to examine the factor structure of the Brief-COPE within a sample of Chinese nursing students. The findings offer valuable insights into the multifaceted nature of coping, emphasizing that coping is a complex, multidimensional process, even when using a general coping scale. Several coping strategies were identified, with some dimensions consistent with previous research, while others reveal distinct coping patterns specific to the study population. For example, a study utilizing the Spanish version of the Brief-COPE identified a 14-factor first-order structural model with robust reliability and satisfactory fit indices among student populations in Spain [22]. Similarly, another Spanish study reported an 11-factor structural model in a student cohort [32]. Among students at the University of Hong Kong, an 11-factor model based on action-oriented goals demonstrated strong model fit across samples [33]. The variations in factor structures observed across studies may reflect differences in coping strategies across populations and contexts. Coping behaviors are inherently dynamic, influenced by individual characteristics, the nature of the stressor, temporal contexts, and environmental factors [34,35,36]. These factors underscore the nature of coping strategies, which are often tailored to the specific demands of the stressor, potentially accounting for the heterogeneity in factor structures across different studies. Nevertheless, prior research frequently converges on identifying eight overarching coping dimensions across diverse populations and settings. For instance, these eight factors have been observed among Romanian youth during the COVID-19 pandemic [37], cancer patients undergoing radiation therapy [38], federally incarcerated individuals in Canada [39], and Spanish adolescents [40]. The recurrence of these eight dimensions across varied demographic groups and situational contexts suggests a degree of consistency and universality in the coping structures captured by the Brief-COPE.

The eight-factor structure of the Brief-COPE reveals diverse and nuanced coping mechanisms employed by Chinese nursing students, offering insights into their adaptive and maladaptive responses to stress. Each factor underscores the interplay of personal, cultural, and situational influences shaping coping behaviors. For positive reframing coping, this factor encompasses planning, active coping, positive reframing, and acceptance, representing a proactive and cognitive approach to stress management. Positive reframing fosters resilience by encouraging individuals to focus on alternative perspectives or positive aspects of stressful situations [41]. In the context of Chinese nursing students, this strategy is particularly effective, as it aligns with the cultural value of perseverance and the belief in overcoming adversity through continuous effort. Planning and active coping are highly relevant in the Chinese educational system, which places significant emphasis on academic achievement and discipline. For nursing students, this strategy enhances problem-solving capabilities and optimism, both of which are critical in high-pressure academic and clinical environments.

Planning and active coping emphasize structured and action-oriented strategies, empowering students to regain a sense of control and competence. In the Chinese educational context, where success is often seen as the result of hard work and diligence, these strategies are especially relevant. The culture values persistence and effort, making proactive coping strategies like planning and active coping particularly effective in helping students manage stress and achieve academic goals [42]. The inclusion of acceptance highlights emotional maturity, enabling students to acknowledge and adapt to situations that cannot be changed. This integrative coping approach aligns with Western models and Chinese cultural values of perseverance and adaptability [43].

Avoidant and passive coping strategies, such as substance use and disengagement, are often associated with negative outcomes, including heightened stress and impaired functioning [44]. However, the inclusion of religion within this factor suggests cultural nuances. In Chinese culture, spiritual practices are often viewed as both sources of solace and avoidance mechanisms. While some nursing students may turn to religious or spiritual practices to avoid confronting stressors, these practices can also offer emotional comfort and a sense of inner peace. This dual role of spiritual practices highlights the complexity of coping mechanisms in the Chinese cultural context, where religious and spiritual beliefs may provide emotional relief but may also delay active engagement with stressors.

Seeking social support reflects the collectivist values deeply ingrained in Chinese society, emphasizing reliance on family, peers, and mentors for emotional and instrumental support [45]. Emotional support helps reduce feelings of isolation, while instrumental support provides practical assistance with academic or clinical tasks. Nursing educators can leverage coping strategies by fostering peer support networks and providing stress-management workshops. Also, offering personalized guidance and promoting self-care practices can help students effectively manage academic and clinical challenges [46].

Self-blame, as a form of emotional distress coping, may undermine self-efficacy and exacerbate psychological distress. This is particularly evident in high-achieving groups, such as nursing students, where perfectionistic tendencies are common [47]. While venting offers temporary relief, it lacks the constructive components necessary for resolving underlying issues. Behavioral disengagement further compounds emotional difficulties by avoiding proactive solutions [48]. This factor highlights the vulnerability of nursing students to emotional distress, underscoring the need for interventions that promote self-compassion and emotional regulation.

Denial, as a defense mechanism, may temporarily alleviate emotional burden but often delays effective problem-solving. Research suggests that, in specific contexts, denial can mitigate anxiety and depression, such as during acute health crises [49]. Humor, on the other hand, functions as a social tool to diffuse tension and maintain harmony [50]. While these strategies may provide immediate comfort, over-reliance on deflection can impede personal growth and adaptation. Interventions for nursing students should aim to balance emotional relief with constructive coping strategies.

Spiritual practices, such as prayer or meditation, offer existential comfort and a sense of purpose, which are particularly valued in Chinese culture [51]. For example, a study in Taiwan evaluated the impact of an elective spiritual education course on nursing students’ spiritual competencies. The results showed that the intervention group experienced significant improvements in spiritual health, practicum stress, professional commitment, spiritual care attitudes, and caring behavior compared to the control group, highlighting the potential benefits of incorporating spiritual education into the nursing curriculum to enhance students’ spiritual competencies and patient care skills [52]. For nursing students, integrating spirituality offers an accessible and culturally resonant approach to emotional well-being.

The last two factors, avoidance and emotional release coping, and adaptive acceptance with distraction, reflect distinct approaches to managing stress. Self-distraction offers temporary relief by allowing individuals to recharge and regain perspective [53]. The effectiveness of distraction is context-dependent; it can help reduce emotional distress in scenarios where stressors are unchangeable [54]. Venting emotions, while providing immediate release, risks perpetuating stress if not followed by constructive action. For nursing students, these findings highlight the need to balance short-term coping strategies with long-term approaches, such as mindfulness or emotional regulation techniques. Acceptance, on the other hand, enables individuals to acknowledge and adapt to stressors that cannot be changed [55]. When combined with self-distraction, it not only offers emotional relief but also fosters resilience. This pragmatic coping style aligns with the dynamic demands of nursing students’ academic and clinical responsibilities. Encouraging such a balanced approach in nursing education could enhance both emotional well-being and professional growth.

### 4.1. Limitations

This study has several limitations. First, our sample consisted exclusively of nursing students, which provides insights into coping strategies within the nursing profession. However, the study focused primarily on students from a single educational institution, limiting the generalizability of the findings to other student populations or students currently in clinical practice. Future research should aim to explore coping strategies across broader populations, including individuals from various healthcare disciplines. Moreover, incorporating students from a variety of institutions would offer a more comprehensive understanding of coping in different environments. Another limitation is the reliance on non-probabilistic sampling, which may constrain the generalizability of the results to the wider population. Recruitment and data collection were conducted online, and the potential homogeneity of the sample may have influenced the study outcomes. Future studies could address this issue by adopting random sampling methods to enhance the representativeness of the findings. Additionally, the cross-sectional design of this study prevented the assessment of the instrument’s stability over time. Longitudinal research would be valuable to determine the temporal consistency of the identified coping strategies. Furthermore, it is crucial for future studies to validate the instrument across diverse, non-university populations to ensure its applicability to various demographic groups.

### 4.2. Implications of Brief-COPE for Nursing

The Brief-COPE is a valuable instrument for evaluating coping strategies among Chinese nursing students. In academic environments, it can help educators identify students experiencing stress and design interventions that promote adaptive coping mechanisms. For example, students who primarily rely on maladaptive strategies, such as denial or self-blame, could benefit from targeted interventions like stress management workshops or counseling to develop more constructive approaches. At the same time, it is equally important for educators to recognize students who demonstrate effective coping strategies, such as problem-solving and seeking support, and reinforce these behaviors to further enhance resilience. In clinical contexts, the Brief-COPE might be used to monitor nursing students’ stress management during clinical placements. Understanding their coping strategies enables clinical educators to provide tailored support, fostering students’ emotional well-being and resilience. Additionally, the tool facilitates tracking changes in coping behaviors over time, allowing educators and healthcare institutions to assess the efficacy of stress management programs and promote the development of both adaptive and effective coping strategies.

### 4.3. Recommendations for Enhancing Coping for Nursing Students

Given the prominence of active coping strategies, such as planning and seeking support, nursing education programs should focus on cultivating problem-solving and time management skills. Workshops and training on academic workload management, effective planning, and seeking appropriate support can empower students to build coping strategies and reduce stress. Nursing programs should also emphasize emotional support by establishing platforms for students to share their experiences and challenges. Peer support groups, mentorship initiatives, and accessible counseling services—led by faculty mentors, peer leaders, or licensed counselors—can provide essential emotional outlets, fostering a supportive academic environment. Furthermore, coping interventions should be culturally attuned to reflect the unique values that influence coping behaviors among Chinese nursing students. For example, promoting strategies aligned with cultural norms, such as seeking support from family and peers, can effectively enhance coping skills.

## 5. Conclusions

The Brief-COPE has been demonstrated to be a valid and reliable instrument for assessing coping strategies among Chinese nursing students. The eight-factor structure highlights the complexity of these strategies, emphasizing the importance of culturally tailored interventions. Nursing educators should leverage interventions to guide students toward appropriate coping strategies, enhancing resilience, improving stress management, and supporting their overall well-being and professional development.

## Figures and Tables

**Table 1:** Results of exploratory factor analysis.

Items of Brief-COPE	Factor							
	1	2	3	4	5	6	7	8
C24 I’ve been learning to live with it.	0.848							
C25 I’ve been thinking hard about what steps to take.	0.802							
C2 I’ve been concentrating my efforts on doing something about the situation I’m in.	0.688							
C7 I’ve been taking action to try to make the situation better.	0.594							
C12 I’ve been trying to see it in a different light, to make it seem more positive.	0.520							
C17 I’ve been looking for something good in what is happening.	0.464							
C14 I’ve been trying to come up with a strategy about what to do.	0.448							
C11 I’ve been using alcohol or other drugs to help me get through it.		0.780						
C4 I’ve been using alcohol or other drugs to make myself feel better.		0.753						
C22 I’ve been trying to find comfort in my religion or spiritual beliefs.		0.541						
C16 I’ve been giving up the attempt to cope.		0.540						
C23 I’ve been trying to get advice or help from other people about what to do.			0.719					
C15 I’ve been getting comfort and understanding from someone.			0.717					
C5 I’ve been getting emotional support from others.			0.665					
C10 I’ve been getting help and advice from other people.			0.447					
C13 I’ve been criticizing myself.				0.753				
C26 I’ve been blaming myself for things that happened.				0.631				
C21 I’ve been expressing my negative feelings.				0.506				
C6 I’ve been giving up trying to deal with it.				0.484				
C3 I’ve been saying to myself “this isn’t real”.					0.732			
C28 I’ve been making fun of the situation.					0.658			
C8 I’ve been refusing to believe that it has happened.					0.572			
C27 I’ve been praying or meditating.						0.546		
C18 I’ve been making jokes about it.						0.507		
C1 I’ve been turning to work or other activities to take my mind off things.							0.844	
C9 I’ve been saying things to let my unpleasant feelings escape.							0.452	
C20 I’ve been accepting the reality of the fact that it has happened.								0.844
C19 I’ve been doing something to think about it less, such as going to movies, watching TV, reading, daydreaming, sleeping, or shopping.								0.452
Variance explained (%)	12.09	10.54	8.39	7.39	6.44	5.01	4.92	4.84

Principal component analysis with Varimax rotation and Kaiser normalization. Kaiser–Meyer–Olkin (KMO) measure of sampling adequacy = 0.82. Bartlett’s Test of Sphericity = 1910.91, *p* < 0.001. Item loadings ≥ 0.40 were shown.

**Table 2:** Internal consistency of the overall scale, eight-factor structure.

	Cronbach’s Alpha	Item–Total Correlation
The whole scale of Brief-COPE	0.759	0.22–0.64
The 8-factor structure		
Factor 1	0.834	0.39–0.67
Factor 2	0.711	0.25–0.55
Factor 3	0.705	0.29–0.51
Factor 4	0.717	0.36–0.41
Factor 5	0.683	0.33–0.59
Factor 6	0.721	0.29–0.38
Factor 7	0.679	0.35–0.44
Factor 8	0.622	0.24–0.31

## Data Availability

The data supporting this study’s findings are available from the corresponding author, upon reasonable request.

## Public Involvement Statement

There was no public involvement in any aspect of this research.

## Guidelines and Standards Statement

This manuscript was drafted against the Strengthening the Reporting of Observational Studies in Epidemiology (STROBE) Statement.
